# Inhibitory effect of luteolin on the metabolism of vandetanib *in vivo* and *in vitro*

**DOI:** 10.3389/fphar.2025.1526159

**Published:** 2025-03-03

**Authors:** Yuxin Shen, Fengsheng Hong, Hualu Wu, Xiaohai Chen, Hailun Xia, Ren-ai Xu, Guanyang Lin, Lu Shi

**Affiliations:** ^1^ The First Affiliated Hospital of Wenzhou Medical University, Wenzhou, Zhejiang, China; ^2^ Zhejiang Key Laboratory of Intelligent Cancer Biomarker Discovery and Translation, First Affiliated Hospital, Wenzhou Medical University, Wenzhou, Zhejiang, China

**Keywords:** vandetanib, luteolin, drug-drug interaction, UPLC-MS/MS, molecular docking

## Abstract

This study aimed to examine the potential drug-drug interaction (DDI) between vandetanib and luteolin *in vivo* and *in vitro*, with the objective of establishing a scientific foundation for their appropriate utilization in clinical settings. Sprague-Dawley (SD) rats were randomly divided into two groups: a control group (vandetanib administered by gavage alone) and an experimental group (vandetanib and luteolin administered together). A series of blood samples were collected at different time intervals. The plasma concentrations of vandetanib and its metabolite N-demethyl vandetanib in rats were determined using an ultra performance liquid chromatography tandem mass spectrometry (UPLC-MS/MS). Incubation systems were set up with rat liver microsomes (RLM) and human liver microsomes (HLM) to measure the Michaelis-Menten constant (K_m_) and half-maximum inhibitory concentration (IC_50_) values. Additionally, the inhibitory mechanism of luteolin on vandetanib was also investigated. Ultimately, the molecular mechanism of inhibition was examined through the utilization of molecular docking techniques. *In vivo* animal experiment results showed that compared with the control group, the AUC_(0-t)_ and C_max_ of vandetanib in the experimental group were significantly increased. The findings from the *in vitro* experiments revealed that luteolin exhibited a moderate inhibitory effect on the metabolism of vandetanib. The IC_50_ values for RLM and HLM were determined to be 8.56 μM and 15.84 μM, respectively. The identified inhibition mechanism was classified as mixed. This study utilized molecular docking analysis to provide additional evidence supporting the competitive inhibition of luteolin on vandetanib in CYP3A4. The data presented in our study indicated a potential interaction between vandetanib and luteolin, which may necessitate the need for dose adjustment during their co-administration in clinical settings.

## 1 Introduction

Vandetanib, an orally administered synthetic compound, demonstrated novel properties as an antagonist by effectively suppressing the activity of vascular endothelial growth factor receptor 2 (VEGFR2), VEGFR3, EGFR, as well as ret tyrosine kinases. These receptors have been implicated in the processes of tumor growth, progression, and angiogenesis (Morabito et al., 2010). On 6 April 2011, vandetanib was received approval for the treatment of medullary thyroid cancer (MTC), marking it as the first targeted therapy authorized by the U.S. Food and Drug Administration (FDA) specifically for this cancer type (Chau and Haddad, 2013; Tsang et al., 2016). Common adverse drug events after vandetanib treatment include diarrhea, rash, fatigue, nausea and hypertension (Grande et al., 2013; Koehler et al., 2021). In cancer chemotherapy, small changes in drug metabolism may affect drug pharmacokinetics, leading to serious clinical consequences. The phase I clinical trial indicated that vandetanib, as a monotherapy at a daily dose of ≤300 mg, demonstrated good tolerability and bioavailability. In the phase II trial, vandetanib exhibited antitumor activity in various malignancies (Morabito et al., 2009; De Luca et al., 2014). Vandetanib was metabolized through Phase I metabolism to generate four metabolites *in vivo*, all of which occur on the N-methyl piperidine part of vandetanib, namely, N-oxidation, N-demethylation, α-hydroxylation and α-carbonyl formation. The generation of metabolites increased the toxicity and instability of vandetanib (Attwa et al., 2018). Research has indicated that the primary cytochrome P450 (CYP450) enzyme responsible for vandetanib metabolism is CYP3A4 (Han et al., 2021). Vandetanib was metabolized to N-demethyl vandetanib by CYP3A4 and converted to vandetanib N-oxide by flavin-containing monooxygenases (FMOs) expressed in the kidney (FMO1) and liver (FMO3). Although N-demethyl vandetanib maintains similar potency to that of vandetanib, vandetanib N-oxide exhibits over 50 times less activity compared to the original compound (Indra et al., 2020).

CYP3A4 was a crucial enzyme involved in drug metabolism within the CYP450 superfamily. It was highly expressed in the human liver and significantly contributed to the metabolism of various drugs (Werk and Cascorbi, 2014). For example, the AUC of vandetanib was significantly decreased when vandetanib was combined with rifampicin, a strong inducer of CYP3A4. Conversely, combining vandetanib with itraconazole, a potent CYP3A4 inhibitor, resulted in increased levels of vandetanib exposure (Martin et al., 2011). Thus, alterations in CYP450 can impact the metabolism of vandetanib.

Luteolin is a tetrahydroxy flavonoid compound that has antioxidant, anti-inflammatory, anticancer and immunomodulatory activities (Du and Shen, 2023). In traditional Chinese medicine, luteolin has been utilized for the treatment of conditions like hypertension, inflammatory diseases, and cancer (Imran et al., 2019). Consequently, luteolin may serve as a complementary treatment to safeguard against and suppress the growth of human cancers. It was found that luteolin could inhibit the growth of thyroid cancer cells by reducing BRAF-activated non-protein coding RNA (BANCR) and thyroid stimulating hormone receptor (TSHR) (Liu et al., 2017; Du and Shen, 2023).

Drug-drug interaction (DDI) was considered to be an important factor leading to differences in plasma drug exposure. Given the potential of luteolin in the treatment of thyroid cancer and the wide application of vandetanib, it was important to study its interaction. Therefore, in this study, an ultra performance liquid chromatography tandem mass spectrometry (UPLC-MS/MS) method was used to determine the concentrations of vandetanib and its active metabolite N-demethyl vandetanib. The interaction between vandetanib and luteolin was studied using rat liver microsomes (RLM), human liver microsomes (HLM) and Sprague-Dawley (SD) rats. Additionally, molecular docking simulation was used to explore the molecular mechanism underlying the impact of luteolin on vandetanib metabolism. We hope that the DDI study of vandetanib and luteolin in this work can provide some references for clinical individualized precision medicine, thereby promoting rational clinical drug use.

## 2 Materials and methods

### 2.1 Chemicals and reagents

Vandetanib, luteolin and regorafenib (used as internal standard, IS) used in the experiment were purchased from Shanghai Perfemiker Chemical Technology Co., Ltd. (Shanghai, China). Nicotinamide adenine dinucleotide phosphate (NADPH) was purchased from Shanghai Aladdin Biochemical Technology Co., Ltd. (Shanghai, China). Methanol and acetonitrile (HPLC-grade) were purchased from Merck (Darmstadt, Germany). Formic acid was purchased from Anaqua Chemicals Supply (ACS, United States). Ultrapure water was from the Milli-Q Water purification system (Millipore, Bedford, United States). RLM was prepared according to the literature (Wang et al., 2015). HLM was purchased from iPhase Pharmaceutical Services Co., Ltd. (Jiangsu, China). All other chemicals and biological products were of analytical grade or above.

### 2.2 Instruments and conditions

The UPLC-MS/MS system had a Waters Acquity UPLC I-Class system (Milford, MA, United States) and a Waters Xevo TQ-S triple quadrupole tandem mass spectrometer (Milford, MA, United States). The Waters Acquity UPLC I-Class was used to separate vandetanib, N-demethyl vandetanib, and IS with an Acquity BEH C18 column (2.1 mm × 50 mm, 1.7 μm) at 40°C. The mobile phase was consisted of 0.1% formic acid (solution A) and acetonitrile (solution B) with a gradient elution at a flow rate of 0.4 mL/min. The total run time was 2.0 min, and the injection volume was 0.5 μL. The temperature conditions were set as follows: the column temperature was 40°C and the autosampler temperature was 10°C.

**FIGURE 1:**
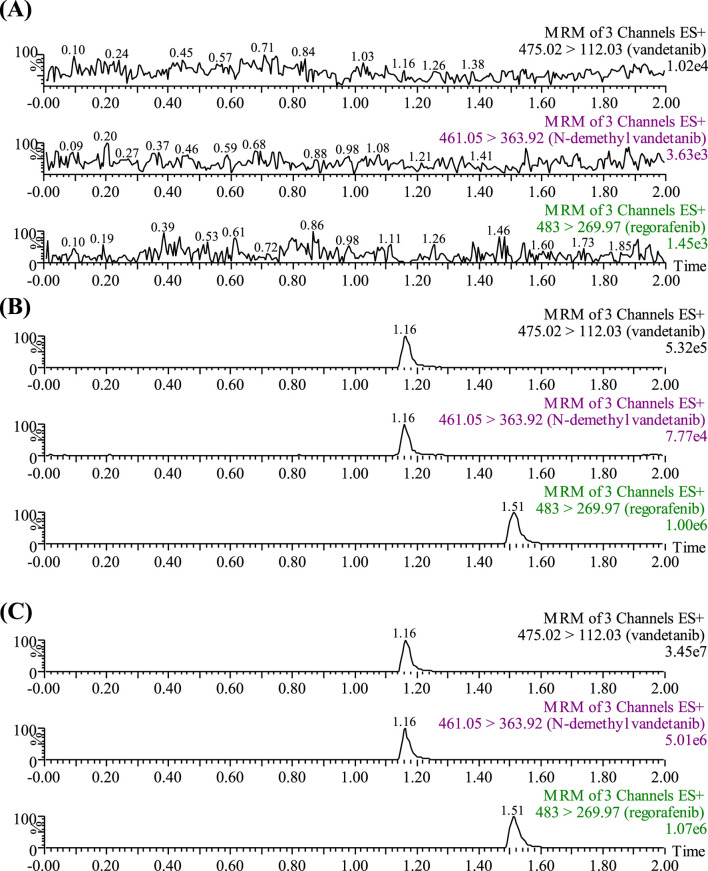
UPLC-MS/MS chromatographs of vandetanib, N-demethyl vandetanib and regorafenib (IS). **(A)** Blank plasma sample, no analyte, no IS. **(B)** Blank rat plasma added with analytes at LLOQ. **(C)** Rat plasma sample after the administration of vandetanib.

A Waters Xevo TQ-S triple quadrupole mass spectrometer was equipped with electrospray ionization (ESI) in positive ion mode, and multiple reaction monitoring (MRM) mode was selected for quantification. The parent and product ions of vandetanib, N-demethyl vandetanib and IS were *m/z* 475.02→112.03, m*/z* 461.05→363.92 and *m/z* 483.00→269.97, respectively. The optimal MS parameters were defined as follows: the cone voltages of vandetanib, N-demethyl vandetanib and IS were set to be 10 V, 10 V and 20 V, respectively, and the collision energies were set to be 10 eV, 10 eV and 30 eV, respectively. Masslynx 4.1 software (Milford, MA, United States) was used for data acquisition, and the chromatograms of analytes were shown in Figure 1.

### 2.3 Method validation

In order to ensure the accuracy and reliability of the analytical methods, we conducted method validation to correctly provide information on the validation parameters. According to the latest guidelines from the FDA, the validation parameters included selectivity, linearity, lower limit of quantification (LLOQ), precision and accuracy, matrix effects, recovery and stability.

### 2.4 Animals and treatment

Male SD rats weighing 180–220 g were provided by the Laboratory Animal Center of the First Affiliated Hospital of Wenzhou Medical University (Zhejiang, China). They were housed under standard conditions with a temperature of 20°C–26°C, a relative humidity of 55% ± 15%, and a 12-h dark/light cycle. The rats had free access to water and no other drugs were given during the feeding period, and were prohibited from eating for 12 h before the experiment. The experimental procedures and protocols were in accordance with animal ethics standards and approved by the Institutional Animal Care and Use Committee of the First Affiliated Hospital of Wenzhou Medical University (Zhejiang, China).

### 2.5 Pharmacokinetics of luteolin on vandetanib *in vivo*

The rats were randomly assigned to two groups: luteolin group (n = 5) and control group (n = 5). Vandetanib and luteolin were both dissolved in 0.5% sodium carboxyl methyl cellulose (CMC-Na) solution. The luteolin group was given luteolin alone (30 mg/kg) by gavage, while the control group was given the same volume of 0.5% CMC-Na solution. 30 min later, each rat was administered of vandetanib (25 mg/kg) by gavage. Regarding the dosage selection for luteolin and vandetanib, we have chosen 30 mg/kg of luteolin and 25 mg/kg of vandetanib based on references from relevant literature (Lin et al., 2014; Yang et al., 2024). Therefore, we adopted the same dosages to ensure the comparability and validity of the experimental results. 0.3 mL tail vein blood was collected at 0.5, 1, 1.5, 2, 3, 4, 6, 8, 12, 24, 48 and 80 h post-vandetanib administration. The blood samples were centrifuged at 4°C at 13,000 rpm for 10 min to obtain plasma, which was aspirated and stored at −80°C until analysis.

### 2.6 *In vitro* effect of luteolin on vandetanib metabolism

Considering that RLM or HLM microsome may have different metabolic rates, we conducted experiments on enzyme and incubation time before the start of the experiment. Regarding the determination of enzyme concentration, we added vandetanib (50 μM), NADPH, potassium phosphate buffer and different concentrations of RLM/HLM in a 200 μL system, controlling the incubation time to 30 min to measure the production of N-demethyl vandetanib. In the incubation time experiment, we determined the concentration of RLM/HLM to be 0.3 mg/mL and compared the production of N-demethyl vandetanib at different incubation times. A rate-time graph was created to select the most suitable concentration and incubation time within the linear range of variation. Taking into account the experimental cost and production yield, we selected an enzyme concentration of 0.3 mg/mL and an incubation time of 30 min. Thus, the total volume of the incubation mixture was 200 μL and was prepared as follows: 0.3 mg/mL RLM (HLM), 100 mM PBS (pH = 7.4), 1 mM NADPH, vandetanib and luteolin. The drug was dissolved in DMSO. The content of organic solvents was less than 1% (Li et al., 2010).

First, in the RLM system, a series of vandetanib at concentrations of 0.1, 0.5, 1, 10, 20, 50, and 100 μM were added to the reaction buffer to determine the Michaelis-Menten constant (K_m_) value. The concentrations of vandetanib in HLM were 1, 10, 50, 100, 300, 800 and 1,000 μM. The relationship between reaction rate and substrate concentration based on the Michaelis-Menten equation was calculated, and the K_m_ value through nonlinear fitting was determined. For half-maximum inhibitory concentration (IC_50_) assay, the concentration gradients of luteolin used were 0, 0.01, 0.1, 1, 10, 25, 50 and 100 μM both in RLM and HLM, and the final concentration of vandetanib was the K_m_ value in the corresponding system. The relationship between inhibitor concentration and reaction rate was plotted after the reaction rate at different inhibitor concentrations was determined, and then the IC_50_ value through nonlinear regression analysis was calculated. Finally, to determine the inhibitory mechanism of vandetanib on luteolin, a series of luteolin concentrations were produced according to the IC_50_ levels, where 0, 2.14, 4.28, 8.56 μM were for RLM and 0, 7.92, 15.84, 31.68 μM were for HLM, respectively. And, the concentrations of vandetanib were set according to the K_m_ value, where the values were 2.35, 4.71, 9.41, 18.82 μM in RLM, and 12.47, 24.94, 49.87, 74.81 μM in HLM, respectively.

The above mixture was incubated at 37°C for 5 min, after which NADPH was added to start the reaction process for 30 min, and it was finally cooled to −80°C to stop the reaction. 400 μL of acetonitrile and 20 μL of IS working solution (200 ng/mL) were incorporated into the mixture. Following vortexed and centrifugated at 13,000 rpm for 10 min, the supernatant was obtained for UPLC-MS/MS analysis.

### 2.7 Molecular docking

The molecular structures of vandetanib, luteolin and itraconazole were obtained from the Pubchem database. PyMOL for structural optimization and AutoDock software for molecular docking verification and binding energy calculation were conducted. Finally, the binding interactions between drugs using PyMOL software was evaluated.

### 2.8 Statistical analysis

K_m_, IC_50_, Lineweaver-Burk plots and mean plasma concentration-time curves were generated using GraphPad Prism 9.5 software. Drug and Statistics (DAS, Chinese Committee of Mathematical Pharmacology, Shanghai, China) was intended to calculate the pharmacokinetic parameters of vandetanib and its metabolite, including time to peak (T_max_), maximum plasma concentration (C_max_), elimination half-life (t_1/2_), area under the drug-time curve (AUC), and clearance (CL_z_/F). SPSS (version 26.0; SPSS Inc., Chicago, IL, United States) was used for data processing, and *t*-test was employed to determine whether there was a significant difference. *P* value <0.05 indicated a significant difference compared to the control group.

## 3 Results

### 3.1 Chromatographic method for vandetanib and its metabolite

Under the chromatographic conditions listed above, the retention times of vandetanib, the metabolite N-demethyl vandetanib, and IS were 1.16 min, 1.16 min and 1.51 min, respectively. As shown in Figure 1, no interference was detected in rat plasma between vandetanib, N-demethyl vandetanib and IS. The calibration curve of vandetanib was linear over the concentration range of 2–1,000 ng/mL with a typical regression equation of Y = 0.0012972 × X + 0.00147,298 (*r* ^2^ = 0.996). And, N-demethyl vandetanib was measured from 0.5 to 50 ng/mL, with a regression equation of Y = 0.00119,975 × X + 0.00104,396 (*r* ^2^ = 0.991). The LLOQ of vandetanib and N-demethyl vandetanib were 2 ng/mL and 0.5 ng/mL, respectively, in this developed UPLC-MS/MS method. The results of accuracy, precision, recovery rate and matrix effect were presented in Supplementary Table S1. Also, the stability results were complied with FDA guidelines. These findings demonstrated that the established method was accurate and dependable.

### 3.2 *In vivo* pharmacokinetic assays

**FIGURE 2:**
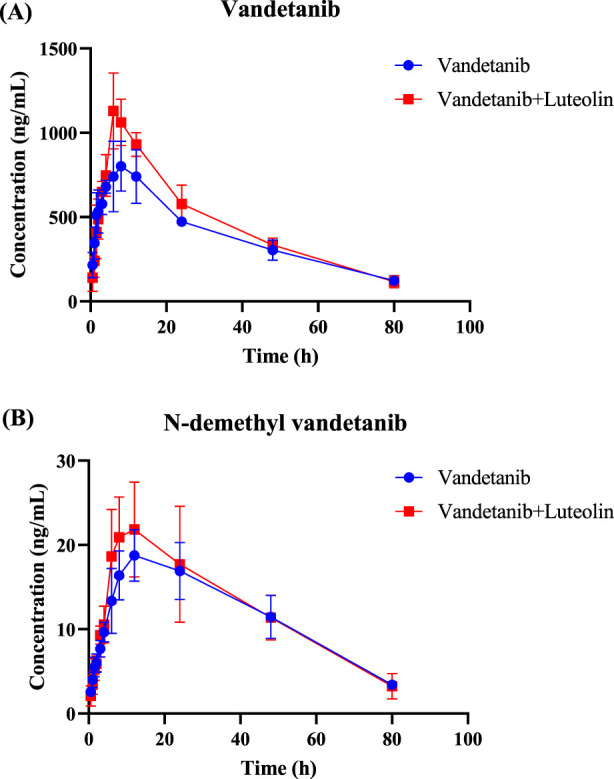
Mean plasma concentration–time curves of vandetanib **(A)** and N-demethyl vandetanib **(B)** in rats.

The mean plasma concentration-time curves of vandetanib and its major metabolite after administration in rats were shown in Figure 2. The detailed pharmacokinetic parameters of vandetanib and N-demethyl vandetanib were shown in Table 1 and Table 2, respectively. In this study, it was found that compared with the control group, the AUC_(0-t)_ of vandetanib in luteolin group was increased by 18.1% and C_max_ was increased by 32.8%, while there were no statistically significant differences in other pharmacokinetic parameters. Moreover, there was no significant change in the metabolite. These data suggested that luteolin could significantly increase the plasma concentration and exposure of vandetanib by inhibiting the metabolism of vandetanib in rats.

**TABLE 1:** The main pharmacokinetic parameters of vandetanib in SD rats.

Parameters	Vandetanib	Vandetanib + Luteolin
AUC_(0-t)_ (μg/L*h)	31,350.29 ± 2,839.70	37,051.06 ± 4,082.07*
AUC_(0-∞)_ (μg/L*h)	36,073.35 ± 4,566.48	41,095.95 ± 4,309.45
t_1/2_ (h)	26.55 ± 4.35	22.99 ± 4.02
T_max_ (h)	8.80 ± 3.03	7.60 ± 2.61
CL_z_/F (L/h/kg)	0.70 ± 0.09	0.61 ± 0.06
C_max_ (μg/L)	859.16 ± 124.58	1,140.87 ± 217.62*

*P < 0.05, in comparison with group vandetanib alone.

**TABLE 2:** The main pharmacokinetic parameters of N-demethyl vandetanib in SD rats.

Parameters	Vandetanib	Vandetanib + Luteolin
AUC_(0-t)_ (μg/L*h)	938.88 ± 144.07	998.82 ± 276.65
AUC_(0-∞)_ (μg/L*h)	1,163.14 ± 157.34	1,118.64 ± 320.66
t_1/2_ (h)	34.04 ± 15.69	24.33 ± 5.63
T_max_ (h)	14.40 ± 5.37	11.20 ± 1.79
CL_z_/F (L/h/kg)	21.83 ± 3.10	23.83 ± 6.50
C_max_ (μg/L)	19.56 ± 3.03	22.41 ± 5.84

### 3.3 Inhibitory effect of luteolin on the metabolism of vandetanib *in vitro*

**FIGURE 3:**
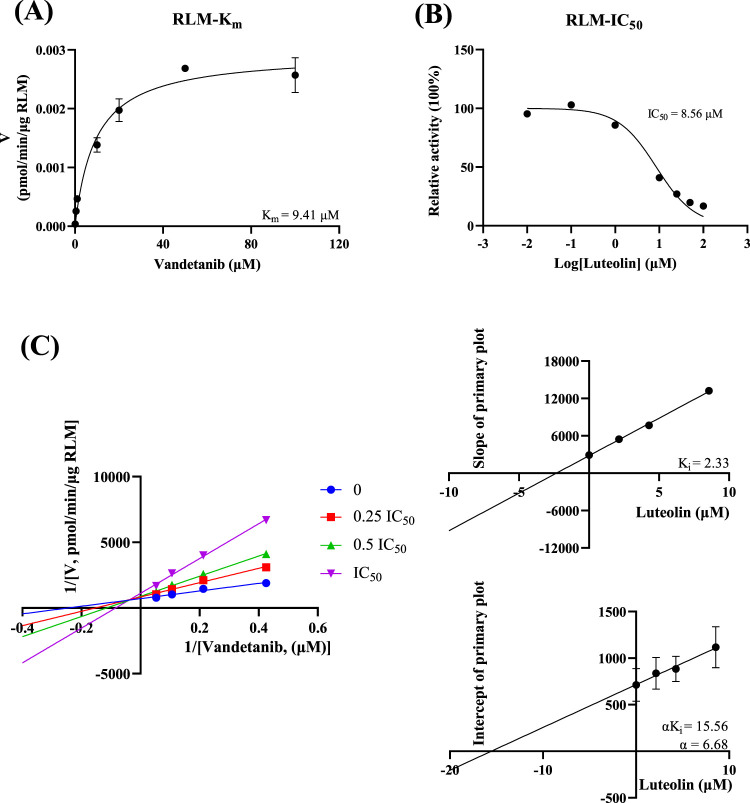
In RLM, Michaelis-Menten plot **(A)**, IC_50_ of luteolin **(B)**, Lineweaver-Burk plot, secondary diagram of K_i_ and secondary diagram of αK_i_ inhibiting vandetanib metabolism at different concentrations of luteolin **(C)** (n = 3).

**FIGURE 4:**
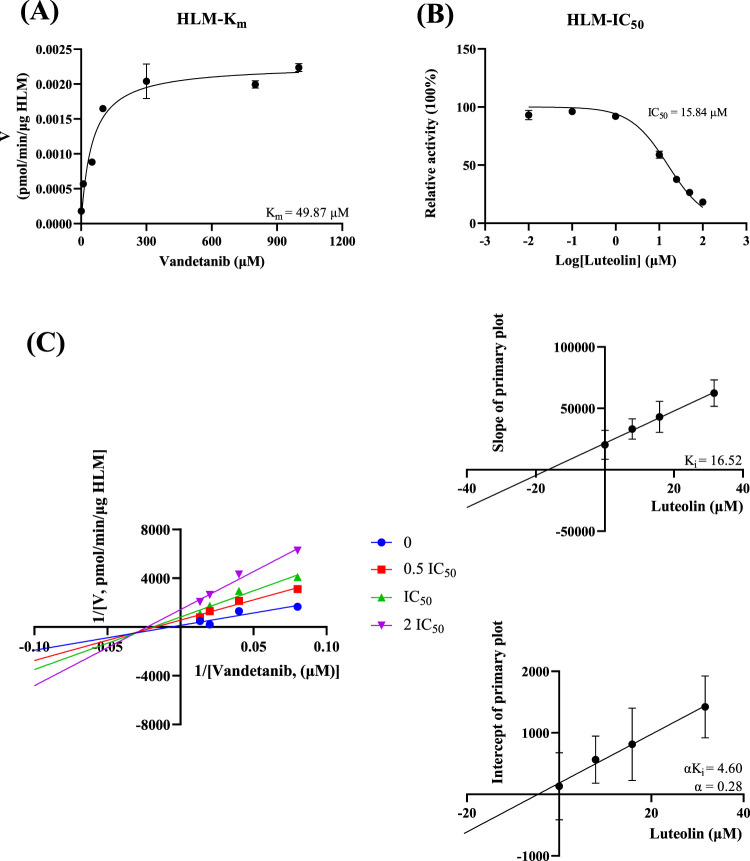
In HLM, Michaelis-Menten plot **(A)**, IC_50_ of luteolin **(B)**, Lineweaver-Burk plot, secondary diagram of K_i_ and secondary diagram of αK_i_ inhibiting vandetanib metabolism at different concentrations of luteolin **(C)** (n = 3).

*In vitro* study, the results showed that the K_m_ of vandetanib was 9.41 μM and the IC_50_ value of luteolin was 8.56 μM in RLM (Figure 3). This suggested that luteolin had an inhibitory effect on vandetanib *in vitro*. The Lineweaver-Burk plot revealed that the inhibition mechanism was a mixture of non-competitive inhibition and competitive inhibition, with the values of K_i_ = 2.33 μM and α = 6.68 μM. As shown in Figure 4, in the HLM incubation system, the K_m_ and IC_50_ values were 49.87 μM and 15.84 μM, respectively. The Lineweaver-Burk plot showed that the inhibition type of luteolin on vandetanib was a mixture of non-competitive and un-competitive, with K_i_ and α values of 16.52 μM and 0.28 μM, respectively.

### 3.4 Molecular docking

**FIGURE 5:**
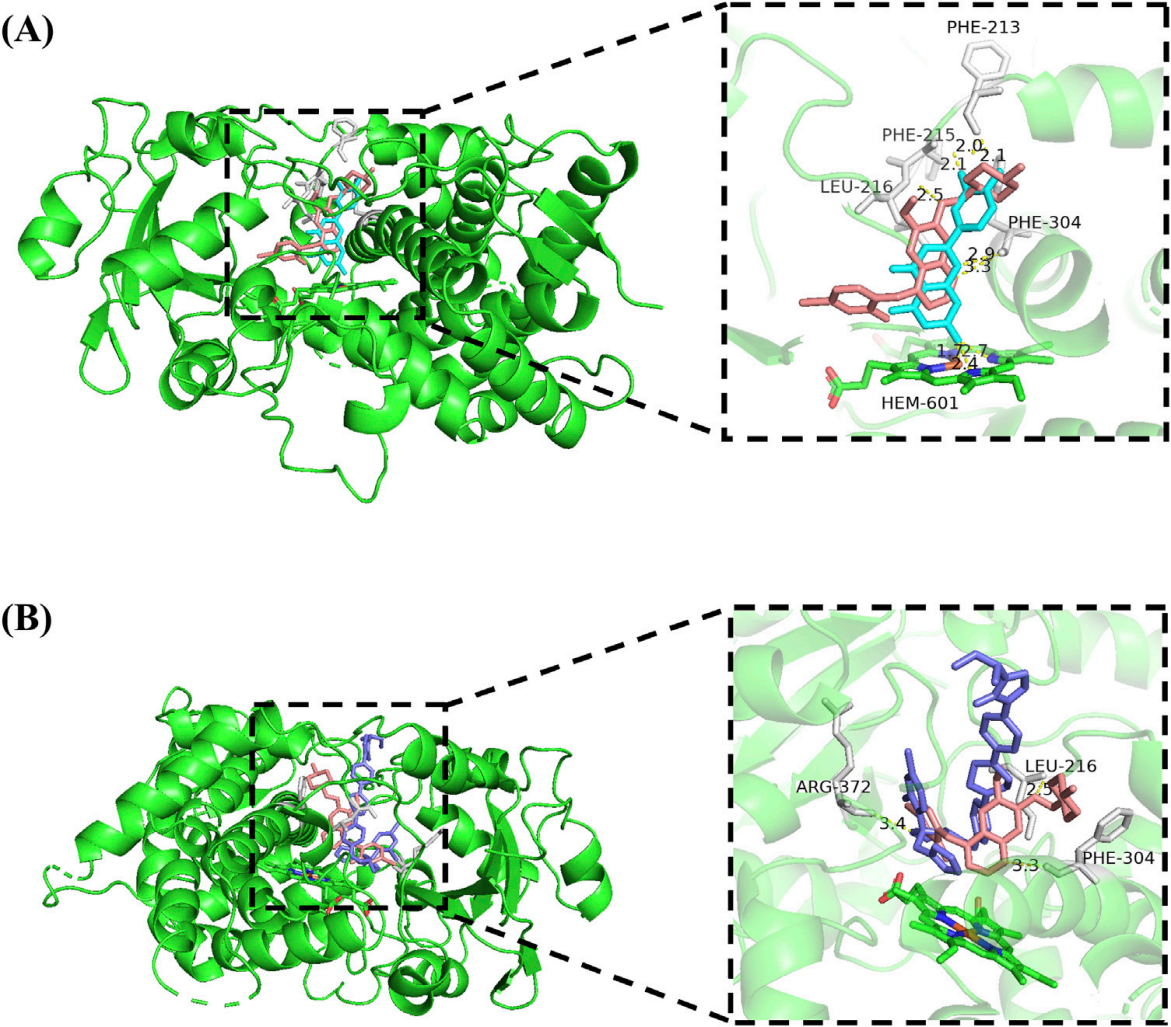
Molecular docking of vandetanib and luteolin with CYP3A4 **(A)**, and molecular docking of vandetanib and itraconazole with CYP3A4 **(B)**. Superimposed 3D structure models with interactions (inset): vandetanib = pink; luteolin = cyan; itraconazole = slate, hydrogen bonding = yellow dashed line.

To gain a deeper insight into the interaction mechanism between vandetanib and luteolin, molecular docking was conducted in this study. The results (Figure 5) showed that vandetanib (pink) formed three hydrogen bonds with residues PHE-304 (3.3 Å), PHE-213 (2.1 Å) and LEU-216 (2.5 Å), and the binding energy was −6.98 kcal/mol. Luteolin (cyan) formed three hydrogen bonds with PHE-304, PHE-213 and LEU-215, with sites 2.9 Å, 2.0 Å and 2.1 Å apart, respectively, and binding energy of −8.46 kcal/mol. We also observed that vandetanib and luteolin have common binding sites: PHE-304 and PHE-213, and that they can spontaneously bind to the active catalytic cavity of CYP3A4. At the same time, we observed a hydrogen bonding interaction between itraconazole (slate) and the active site residue ARG-372 (3.4 Å) that stabilized CYP3A4. The binding energy of itraconazole to CYP3A4 was −5.44 kcal/mol.

## 4 Discussion

Thyroid cancer is a ubiquitous malignancy and its incidence has increased significantly in recent decades (Seib and Sosa, 2019). There are various types of thyroid cancer, with MTC being a relatively rare subtype, representing approximately 1%–2% of all thyroid cancers (Angelousi et al., 2022; Fugazzola, 2023). Despite its rarity, MTC accounts for a significant portion of thyroid cancer-related fatalities, making up about 15% of all deaths associated with thyroid cancer, and has a five-year survival rate of less than 40% (Bhoj et al., 2021; Green et al., 2022). Currently, some tyrosine kinase inhibitors (TKIs) have emerged as promising therapies for MTC that can induce clinical responses and stabilize diseases, such as cabozantinib, vandetanib, selpercatinib (Kim and Kim, 2021; Fallahi et al., 2022). Vandetanib has been approved by the FDA as the preferred option for patients with recurrent or persistent MTC who are not candidates for surgery and whose disease causes symptoms or growth (Haddad et al., 2022). The research indicated that for both long-term and short-term treatment, vandetanib has low toxicity and good efficacy, leading to significant improvements in the quality of life for patients (Tsang et al., 2016; Kreissl et al., 2020; Koehler et al., 2021; Ramos et al., 2021). Additionally, the role of vandetanib in treating children should not be overlooked, as it has been used to treat diffuse intrinsic pontine glioma in children (Bai et al., 2011; Carvalho et al., 2022). Vandetanib was mainly metabolized by CYP3A4, resulting in the formation of the active metabolite N-demethyl vandetanib (Martin et al., 2012; Beaton et al., 2022).

Luteolin was a common flavonoid with a range of pharmacological properties, including anti-inflammatory, neuroprotective, antibacterial, antiviral, and anti-diabetic properties (Rocchetti et al., 2023). At the same time, it made a variety of cancer cells sensitive to treatment-induced cytotoxicity by inhibiting cell survival pathways and stimulating apoptosis pathways, and was a widely used anticancer agent (Lin et al., 2008; Rakoczy et al., 2023). Since the anticancer effects of luteolin look very promising, its potential DDI must be considered (Galati and O'Brien, 2004). It has been reported that flavonoids may have drug interactions when administered in combination, affecting the therapeutic effects of other drugs (Khan et al., 2021). Therefore, additional studies and clinical trials are necessary to validate the safety of the combination of vandetanib and luteolin.

In this study, we developed and validated a sensitive, specific, rapid, and reliable UPLC-MS/MS method for the quantitative analysis of vandetanib and its metabolite. This method had been used for subsequent *in vivo* and *in vitro* studies. In our study, luteolin showed inhibitory effect on vandetanib metabolism. The pharmacokinetic parameters of animal experiments showed that compared with the single administration of vandetanib, the AUC_(0-t)_ and C_max_ of vandetanib were increased in the combined administration group. There was no significant statistical difference in the parameters of the metabolite N-demethyl vandetanib. This means that luteolin inhibited the metabolism of vandetanib in rats, leading to increased drug exposure and adverse reactions. The *in vivo* results were consistent with subsequent *in vitro* results, where luteolin inhibited the metabolism of vandetanib in a mixed manner. It had been reported in the literature that when luteolin was co-administered with drugs metabolized through CYP3A, it may cause pharmacokinetic interactions (Quintieri et al., 2008). Therefore, we speculated that the inhibitory effect of luteolin on vandetanib was mainly through CYP3A4.

*In vitro* enzyme kinetics studies, IC_50_ values told us that luteolin moderately inhibited vandetanib metabolism. Subsequently, we further explored the potential enzymatic and molecular inhibition mechanisms of luteolin on vandetanib metabolism. We found that luteolin inhibited the N-demethylation of vandetanib in a mixed manner in both RLM and HLM.

Hydrogen bonding is not only important for the energetic stabilization of protein structures, but also plays a crucial influence in drug binding affinity (Patil et al., 2010). For this reason, we investigated the docking simulations and weak intermolecular interactions of vandetanib, luteolin, and a strong CYP3A4 inhibitor (itraconazole). The results showed that luteolin and vandetanib were jointly bound to the same site of CYP3A4, which indicated that luteolin competitionally inhibited drug metabolism, and the spatial proximity may be one of the reasons for the relatively easy interaction between the two drugs. Compared to the strong CYP3A4 inhibitor itraconazole, luteolin exhibited a stronger binding ability to CYP3A4. Thus, this somewhat confirmed the inhibitory potential of luteolin on CYP3A4.

To conclude, our results implied that luteolin inhibited the metabolism of vandetanib, which may lead to potential DDI and provide early warning for drug co-use. But it is worth noting that luteolin was a natural compound widely present in various foods, such as celery, green peppers, carrots, onions, broccoli, and more (Yan et al., 2014; Çetinkaya and Baran, 2023). This makes it have important nutritional value and biological activity in our daily diet. However, due to the possible interaction between luteolin and some drugs, we need to pay special attention to the interaction between these foods and drugs when consuming foods containing luteolin (Wang et al., 2008; Liu and Li, 2024). This interaction may alter the absorption, metabolism, and efficacy of the drug, which can affect treatment effectiveness or increase the risk of adverse reactions. Therefore, we should be cautious in our diet and medication use, and try to avoid drug interactions as much as possible. Our research has certain reference value.

## 5 Conclusion

In summary, luteolin significantly inhibited the metabolism of vandetanib. The combination of luteolin and vandetanib resulted in significant increases in AUC_(0-t)_ and C_max_ of vandetanib. The occurrence of such DDI may lead to increased incidence and severity of adverse drug events. To reduce risks, clinical recommendations suggest avoiding the combination of two drugs as much as possible.

## Data Availability

The original contributions presented in the study are included in the article/Supplementary Material, further inquiries can be directed to the corresponding authors.
