# Supplementary material for: Inhibitory effect of luteolin on the metabolism of vandetanib in vivo and in vitro
Source: Front Pharmacol. 2025 Mar 3;16:1526159. doi: 10.3389/fphar.2025.1526159 (PMC11911205; doi:10.3389/fphar.2025.1526159)
Supplement: Supplementary file 1 [file DataSheet1.docx]

**Supplementary Table 1.** Precision and accuracy of vandetanib and its metabolite N-demethyl vandetanib in rat plasma (n = 5).

| Analytes | Concentration  (ng/mL) | Intra-day | | Inter-day | |
| --- | --- | --- | --- | --- | --- |
|  |  | RSD% | RE% | RSD% | RE% |
| Vandetanib | 2 | 8.1 | 1.2 | 14.4 | -5.0 |
|  | 5 | 2.8 | 8.6 | 9.5 | 0.4 |
|  | 400 | 8.7 | 2.2 | 7.5 | 4.6 |
|  | 800 | 9.9 | -0.2 | 8.5 | 3.5 |
| N-demethyl vandetanib | 0.5 | 8.4 | -0.8 | 11.2 | -3.2 |
|  | 1 | 5.6 | 2.9 | 12.2 | -3.0 |
|  | 10 | 8.2 | 5.7 | 7.3 | 5.0 |
|  | 20 | 7.6 | 3.3 | 7.3 | 4.9 |

**Supplementary Table 2.** Recovery and matrix effect of vandetanib and N-demethyl vandetanib in rat plasma (n = 5).

| Analytes | Concentration  (ng/mL) | Recovery (%) | | Matrix effect (%) | |
| --- | --- | --- | --- | --- | --- |
|  |  | Mean ± SD | RSD (%) | Mean ± SD | RSD (%) |
| Vandetanib | 5 | 93.0 ± 9.2 | 9.9 | 105.6 ± 8.3 | 7.8 |
|  | 400 | 97.4 ± 3.4 | 3.5 | 89.6 ± 2.2 | 2.5 |
|  | 800 | 97.1 ± 5.1 | 5.3 | 89.0 ± 2.4 | 2.7 |
| N-demethyl vandetanib | 1 | 86.3 ± 6.3 | 7.3 | 93.2 ± 10.9 | 11.7 |
|  | 10 | 89.4 ± 7.4 | 8.3 | 86.1 ± 4.7 | 5.4 |
|  | 20 | 93.1 ± 3.9 | 4.2 | 101.4 ± 1.5 | 1.5 |
